# Nonsense mutation in *TMEM126A* causing autosomal recessive optic atrophy and auditory neuropathy

**Published:** 2010-04-13

**Authors:** Esther Meyer, Michel Michaelides, Louise J. Tee, Anthony G. Robson, Fatimah Rahman, Shanaz Pasha, Linda M. Luxon, Anthony T. Moore, Eamonn R. Maher

**Affiliations:** 1 Department of Medical and Molecular Genetics, Institute of Biomedical Research, University of Birmingham, Birmingham, UK; 2 UCL Institute of Ophthalmology, 11-43 Bath Street, London, UK; 3 Moorfields Eye Hospital, City Road, London, UK; 4 UCL Ear Institute, 332 Grays Inn Road, London, UK; 5 Great Ormond Street Hospital for Children, Great Ormond Street, London, UK

## Abstract

**Purpose:**

To define the phenotype and elucidate the molecular basis for an autosomal recessively inherited optic atrophy and auditory neuropathy in a consanguineous family with two affected children.

**Methods:**

Family members underwent detailed ophthalmologic, electrophysiological, and audiological assessments. An autozygosity mapping strategy using high-density single nucleotide polymorphism microarrays and microsatellite markers was used to detect regions of genome homozygosity that might contain the disease gene. Candidate genes were then screened for mutations by direct sequencing.

**Results:**

Both affected subjects had poor vision from birth and complained of progressive visual loss over time. Current visual acuity ranged from 6/60 to 6/120. Fundus examination revealed bilateral temporal optic nerve pallor in both patients with otherwise normal retinal findings. International-standard full-field electroretinograms were normal in both individuals, with no evidence of generalized retinal dysfunction. Pattern cortical visual evoked potentials were grossly abnormal bilaterally in both cases. The pattern electroretinogram N95:P50 ratio was subnormal, and the P50 was of shortened peak time bilaterally in both patients. The electrophysiological findings were consistent with bilateral retinal ganglion cell/optic nerve dysfunction. Audiological investigation in both siblings revealed abnormalities falling within the auditory neuropathy/dysynchrony spectrum. There were no auditory symptoms and good outer hair cell function (as demonstrated by transient evoked otoacoustic emissions) but impaired inner hair cell/neural function with abnormal stapedial reflex thresholds and abnormal or absent auditory brainstem-evoked responses. The single nucleotide polymorphism microarray data demonstrated a 24.17 Mb region of homozygosity at 11q14.1–11q22.3, which was confirmed by microsatellite marker analysis. The candidate target region contained the transmembrane protein 126A (*TMEM126A*) gene, and direct sequencing identified a previously described nonsense mutation (c.163C>T; p.Arg55X).

**Conclusions:**

We describe the first detailed phenotyping of patients with autosomal recessive *TMEM126A*-associated optic atrophy and auditory neuropathy. These findings will facilitate the identification of individuals with this recently described disorder.

## Introduction

Primary hereditary optic neuropathies comprise a group of disorders that are characterized by visual loss due to retinal ganglion cell death. The most common forms of optic neuropathy are Leber hereditary optic neuropathy (LHON) with mitochondrial transmission (OMIM 535000) and autosomal dominant optic atrophy (OMIM 165500) [1]. Autosomal recessive optic neuropathies are uncommon and are mostly observed in association with multisystem diseases. A few cases of isolated autosomal recessive optic atrophy have been reported [2]. Previously Barbet et al. [3] mapped a locus for early onset but slowly progressive optic neuropathy (OPA6; OMIM 258500) to chromosome 8q. Affected family members presented with visual impairment commencing between 2 and 6 years of age, moderate photophobia, and dyschromatopsia. There were no associated systemic features. Recently, Hanein et al. [4] identified a second locus for autosomal recessive optic atrophy on chromosome 11 and identified germline mutations in transmembrane protein 126A gene (*TMEM126A*) in affected individuals from four families.

Autosomal recessive auditory neuropathy has been reported in association with mitochondrial myopathy and mitochondrial DNA multiple deletions [5], but commonly it presents as congenital nonsyndromic hearing impairment as a consequence of mutations in the otoferlin (*OTOF*) gene, a membrane-anchored calcium-binding protein that plays a role in the exocytosis of synaptic vesicles at the auditory inner hair cell ribbon synapses [6]. Nonsyndromic autosomal recessive auditory neuropathy has also been reported in association with missense mutations in the autosomal recessive deafness 59 gene on chromosome 2q31.1-q31.3, which encodes the protein pejvakin found in hair cell, supporting cells, spiral ganglion cells, and the first three relays of the afferent auditory pathway [7].

The co-occurrence of optic neuropathy and auditory neuropathy is rare, but two cases of LHON with auditory neuropathy have been reported [8], although a more recent study has documented that this is an uncommon finding in LHON [9]. The X-linked recessive deafness-dystonia-optic neuronopathy syndrome (Mohr-Tranebjaerg syndrome; OMIM 304700) is characterized by postlingual sensorineural hearing loss in early childhood, with progressive neural degeneration affecting the brain, eighth cranial nerve, and optic nerves in adult life. The auditory findings indicate auditory neuropathy, with spiral ganglion cells being the suspected site of pathology. The X-linked recessive deafness-dystonia-optic neuronopathy is caused by mutations in the translocase of inner mitochondrial membrane 8 homolog A (yeast) gene which is also called deafness/dystonia peptide gene and encodes for a 97 amino acid polypeptide [10].

We report the results of detailed clinical, electrophysiological, audiological, and molecular genetic investigations in a family with optic atrophy associated with a mutation in *TMEM126A*. Our findings suggest that auditory neuropathy may be an additional previously unreported feature of this disorder.

## Methods

### Patients

A consanguineous family of Algerian origin with two affected children was ascertained and recruited for clinical and molecular genetic studies. All subjects gave written informed consent. The study was approved by the South Birmingham Local Research Ethics Committee and was performed in accordance with the Declaration of Helsinki. Genomic DNA from the two affected individuals, two unaffected siblings, and the parents were extracted from peripheral lymphocytes by standard techniques.

### Ocular assessment

Both affected siblings (IV:1 and IV:2) were examined. A medical and ophthalmic history was taken, and a full ophthalmologic examination was performed. Color vision was tested using Ishihara pseudoisochromatic plates and Hardy, Rand and Rittler plates (American Optical Company, New York, NY). Each patient underwent color fundus photography, Goldmann perimetry (Haag-Streit AG, Bern, Switzerland), and retinal nerve fiber layer analysis using the Zeiss Stratus® OCT 3 (Carl Zeiss Meditec Inc., Dublin, CA). Both patients had detailed electrophysiological assessment, including a full-field electroretinogram (ERG) and pattern ERG, incorporating the protocols recommended by the International Society for Clinical Electrophysiology of Vision [11,12]. Cortical visual evoked potentials (VEPs) were recorded to high contrast checkerboard reversal (field size 12°×15° or 24°×30°, check size 0.9°, reversal rate 2.2 Hz) and diffuse flash stimulation.

### Audiological assessment

Both siblings underwent a detailed otological examination and showed normal tympanic membranes and tuning fork tests. Standard air conduction pure tone audiometry [13] was conducted in a sound-treated booth, using a GSI 61 audiometer (Model 61; Grason Stadler Inc., Eden Prairie, MN) and TDH 39 headphones (Interacoustic A/S, Assens, DK).

Single frequency tympanometry was performed with a probe signal, an 85 dB sound pressure level (SPL) continuous tone at 226 Hz [13], using a GSI 33 Tympanometer (Model 33; Grason Stadler Inc.). Stapedial reflex thresholds were measured both ipsilaterally and contralaterally, using the method described by Cohen and Prasher [14].

Transient evoked otoacoustic emissions were recorded from each ear using an Otodynamics ILO92 analyzer (Otodynamics Ltd., Hartfield, UK) [15]. A standard nonlinear click stimulus of 80 µs duration was presented at a repetition rate of 50 Hz and an intensity of 80 (±3) dB SPL. The response was averaged over 260 acquisitions, and the total (mean) nonlinear transient evoked otoacoustic emissions (TEOAE) response amplitude (dB SPL) was analyzed.

Brainstem-evoked potentials were recorded using a Medelec Sensor ST10. Standard electroencephalogram (EEG) silver/silver chloride disc electrodes were attached to each mastoid process-A1 and A2 and to the vertex-Cz. Electrode impedance was less than 5 kΩ. An alternating polarity click stimulus of 100 µs electrical duration at an intensity of 90 dBHL was presented via TDH-39 headphones at a repetition rate of 10 Hz. Broadband noise at 50 dBHL was used in the contralateral ear. The analysis was confined to latencies and interwave latencies of waves I, III, and V. The analysis of the conduction latencies was considered abnormal if the value exceeded 2 standard deviations (SDs) from the normal mean or if the responses were unrepeatable or absent. Absolute interaural wave V latencies were also analyzed and were considered to be abnormal if the latency difference of wave V was greater than 2 standard deviations from the normal mean.

### Linkage analysis

A genome-wide linkage scan using Affymetrix 250K Sty1 single nucleotide polymorphism (SNP) mapping arrays according to the manufacturer’s instruction (Affymetrix, Inc., Santa Clara, CA) was undertaken in all siblings to identify shared regions of homozygosity (>2 Mb) in the affected individuals. Briefly, the DNAs (250 ng each) were first digested with Sty1 restriction enzyme (New England Biolabs, Boston, MA) and then ligated to adaptors. Each Sty1 adaptor-ligated DNA was amplified in three 100 µl PCR reactions using AmpliTaq Platinum (Clontech Laboratories, Inc., Palo Alto, CA). Fragmented PCR products were then labeled, denatured and hybridized to the array following washing and staining steps on the Affymetrix GeneChip fluidics station 450. Fluorescence intensities were quantified with an Affymetrix array scanner 3000–7G and the data were collected by the Affymetrix GeneChip Operating Software (GCOS) v 1.4. Genotypes were generated using the GTYPE software for BRLMM analysis using default settings. To evaluate common homozygous regions, microsatellite markers were typed in all family members. Information on primer sequences and the physical location of the markers was obtained from the NCBI database and from the UCSC browser, respectively. Amplification conditions were an initial denaturation of 94 °C for 3 min, followed by 28 cycles of 30 s denaturation at 94 °C, 30 s annealing at 55 °C, and 30 s extension at 72 °C with a final extension at 72 °C for 5 min. The amplified fragments were detected by an automated ABI 3730 DNA Analyzer and analyzed with Genemapper v3.0 software (Applied Biosystems Inc., Foster City, CA).

### Mutational analysis

The family members were screened for mutations in *TMEM126A* by direct sequencing. The genomic DNA sequence of this gene was taken from Ensembl, and primer pairs for the translated exons were designed using primer3 software . The exons were amplified by PCR using BioMix™ Red (Bioline Ltd., London, UK). Amplification conditions were an initial denaturation of 95 °C for 5 min, followed by 35 cycles of 30 s denaturation at 95 °C, 1 min annealing at 60 °C, and 1 min extension at 72 °C with a final extension at 72 °C for 5 min. PCR products were cleaned up with MicroCLEAN (Web Scientific, Crewe, UK) and were directly sequenced by the BigDye Terminator Cycle Sequencing System using ABI PRISM 3730 DNA Analyzer (Applied Biosystems Inc.). DNA sequences were analyzed using Chromas software.

## Results

### Clinical findings

**Figure 1:**
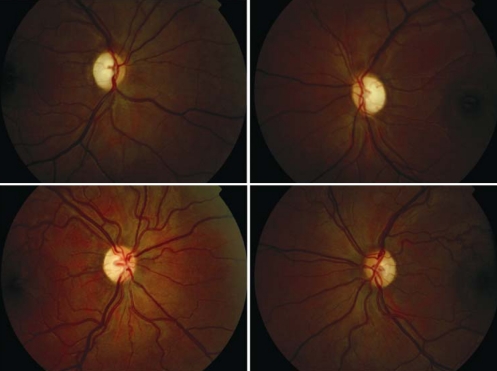
Fundal appearance. Color fundus photographs of both eyes of the two affected siblings. Bilateral temporal optic disc pallor and normal retinal appearance (IV:1 above and IV:2 below) are seen.

**Figure 2:**
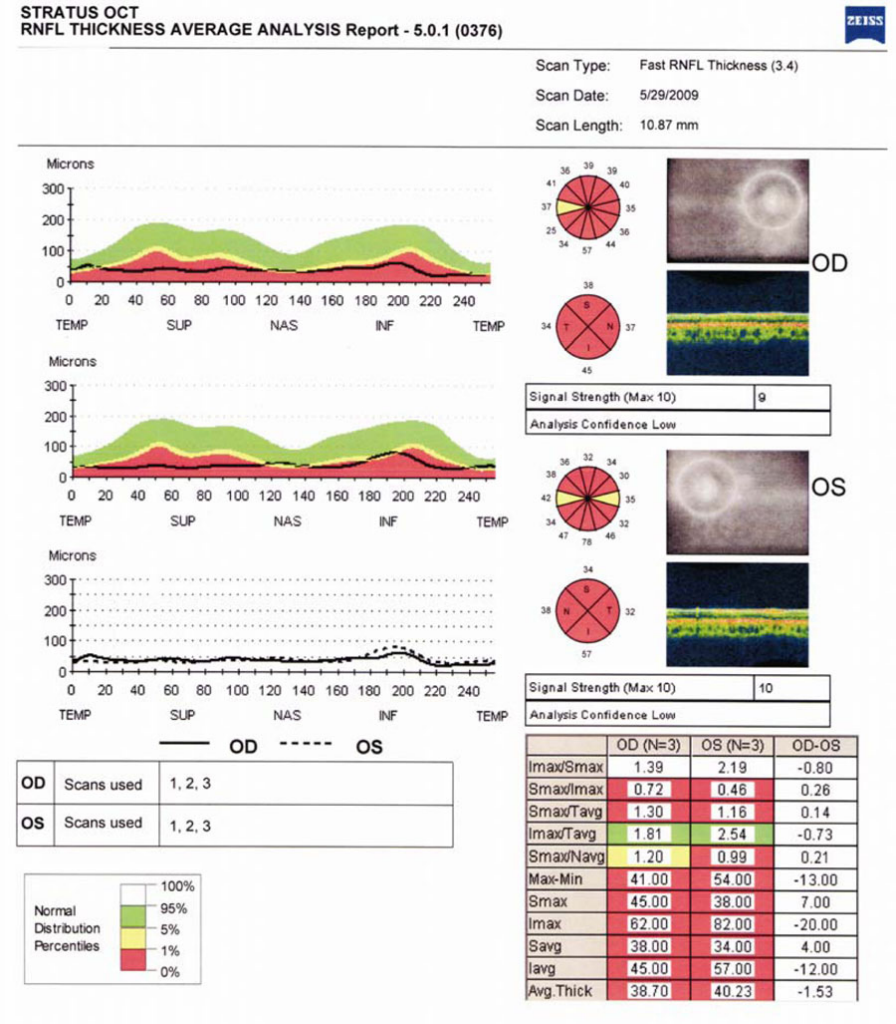
Retinal nerve fiber layer analysis. The results of this analysis demonstrates the marked global reduction in nerve fiber layer thickness compared to normative values (using the Zeiss Stratus® OCT 3) in Patient IV:1.

The ophthalmologic findings of the two affected siblings are summarized in Table 1. Both subjects had poor vision from birth and complained of a continued gradual reduction in vision over time. Patient IV:2 had evidence of a right exotropia and bilateral horizontal nystagmus. Neither patient had any evidence of residual color vision. Examination of the anterior segment was normal in both patients. Fundus examination revealed marked bilateral temporal optic nerve pallor in both patients with otherwise normal retinal findings (Figure 1). Goldmann perimetry identified bilateral visual field constriction in both patients. Retinal nerve fiber layer analysis revealed bilateral marked reduction in nerve fiber layer thickness in both subjects (Patient IV:1—average thickness 38.7 μm right eye, 40.2 μm left eye; range 41.0–54.0 μm. Patient IV:2—average thickness 40.7 μm right eye, 47.3 μm left eye; range 44.0–49.0 μm; Figure 2).

**Table 1:** Summary of ophthalmological findings.

**Patient**	**Sex**	**Age**	**Symptoms**	**Presenting visual acuity OD-OS**	**Current visual acuity OD-OS**	**Refraction**	**Fundus**	**ERG**	**PERG**	**Pattern and flash VEPs**
IV:1	F	19	Reduced vision since birth	3/36–3/36 (14y)	6/95–6/95	−0.75/-0.25x180 −0.75/-0.25x180	Bilateral marked temporal optic nerve pallor	Normal	N95:P50 ratio is subnormal and P50 is of short peak time bilaterally	Severely abnormal pattern and flash VEPs bilaterally.
IV:2	M	17	Reduced vision since birth; horizontal nystagmus; right exotropia	6/60 – 6/60 (12y)	6/120–6/60	−0.75/-0.50x90 plano/-0.50x15	Bilateral temporal optic nerve pallor	Normal	N95:P50 ratio is subnormal and P50 is of short peak time bilaterally	Severely abnormal pattern VEPs. Flash VEPs within normal limits.

Abbreviations: OD-OS represents: Oculus Dexter - Oculum Sinister; ERG represents Electroretinogram; PERG represents Pattern ERG; VEP represents Visual Evoked Potential; F represents Female; M represents Male; y represent Year.

**Figure 3:**
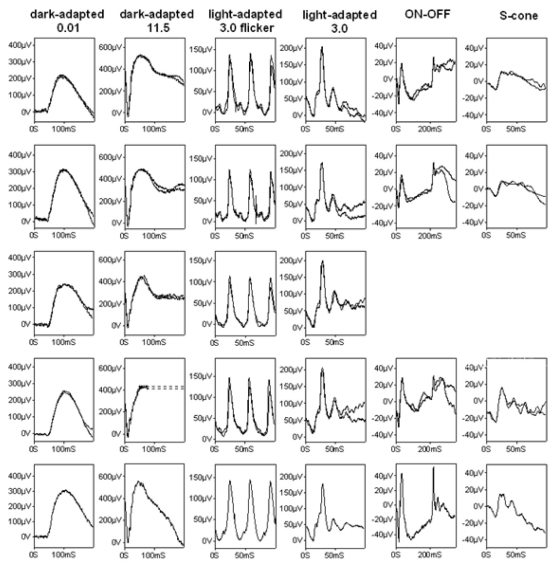
International-standard full-field electroretinogram of the two affected individuals of the optic atrophy family. This figure shows the electroretinogram (ERGs) from the right (row 1) and left (row 2) eye of patient IV:2, from the right (row 3) and left (row 4) eye of patient IV:1, and typical normal examples for comparison (row 5, bottom row). Dark-adapted ERGs are shown for flash intensities of 0.01 and 11.5 cd seconds per square meter (cd.s.m^−2^); light adapted ERGs are shown for 30 Hz flicker and 2 Hz stimulation at a flash intensity of 3.0 cd.s.m^−2^. ON-OFF ERGs used an orange stimulus (560 cds per square meter [cd.m^−2^], duration 200 ms) superimposed on a green background (150 cd.m^−2^). S-cone ERGs used a blue stimulus (445 nm, 80 cd.m^−2^) on an orange background (620 nm, 560 cd.m^−2^). Broken lines replace blink artifacts occurring just after the b-wave peak in patient IV:1.

**Figure 4:**
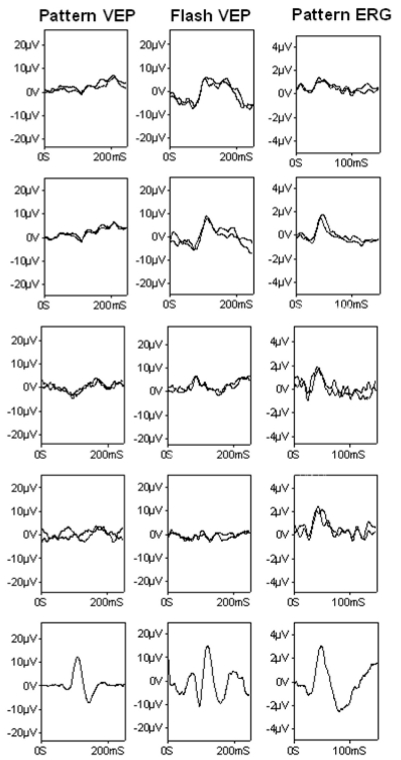
Pattern cortical visual evoked potentials, flash cortical visual evoked potentials, and pattern electroretinograms of the two affected individuals with mutation in the transmembrane protein 126A gene. In row 1 are the results from the right and in row 2 from the left eye of patient IV:2, in row 3 from the right and in row 4 from the left eye of patient IV:1, and in row 5 and the bottom row from normal examples for comparison. Illustrated pattern cortical visual evoked potentials (VEPs) from patient IV:2 were recorded to a large checkerboard field (24°×30°); those from patient IV:1 were recorded to a standard checkerboard field (12°×15°).

Full-field ERGs were normal in both patients, with no evidence of generalized retinal dysfunction (Figure 3). Pattern reversal VEPs from both patients were grossly abnormal (Figure 4). In patient IV:2, pattern VEPs recorded to a large checkerboard field were delayed with an abnormal waveform bilaterally (Figure 4). Pattern VEPs to a standard checkerboard were undetectable on the right, with only a delayed residual component on the left (data not shown). In patient IV:1, pattern VEPs were recorded to a standard checkerboard and were grossly abnormal. Flash VEPs in patient IV:1 had an abnormal waveform of low amplitude bilaterally and revealed no definite abnormality in patient IV:2 (Figure 4). The pattern ERG N95:P50 ratio was subnormal, and the P50 was of short peak time bilaterally in both patients (Figure 4). These VEP and pattern ERG abnormalities are consistent with bilateral retinal ganglion cell/optic nerve dysfunction.

**Figure 5:**
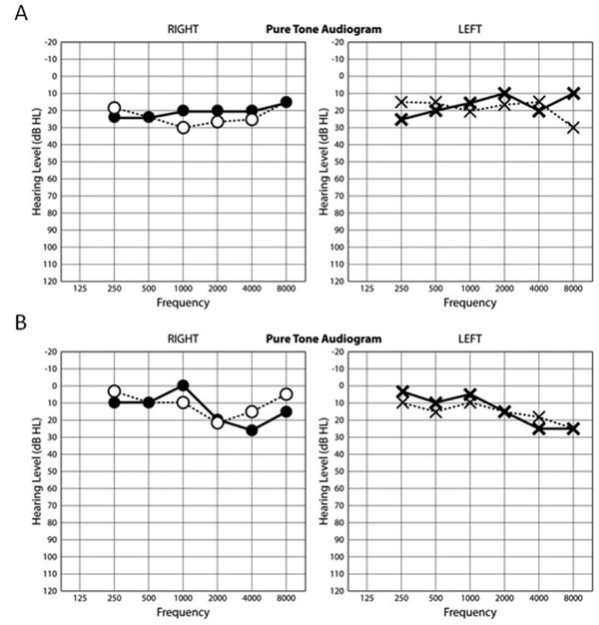
Pure tone audiometry. Pure tone audiometry (250–8000 Hz) in the two affected siblings. The initial recording is shown as a dashed line, and the second recording (about 1 year later) as a solid line. **A**: This panel shows pure tone audiometric thresholds of over a 1-year period of patient IV:1. **B**: This panel shows pure tone audiometric thresholds of over an 18-month period of patient IV:2.

**Figure 6:**
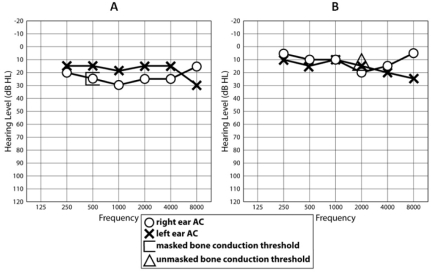
Pure tone audiograms of patient IV:1 (**A**) and patient IV:2 (**B**) to show absence of air-bone gap (i.e., conductive hearing loss), as judged by masked and unmasked bone conduction thresholds. AC represents air conduction.

**Figure 7:**
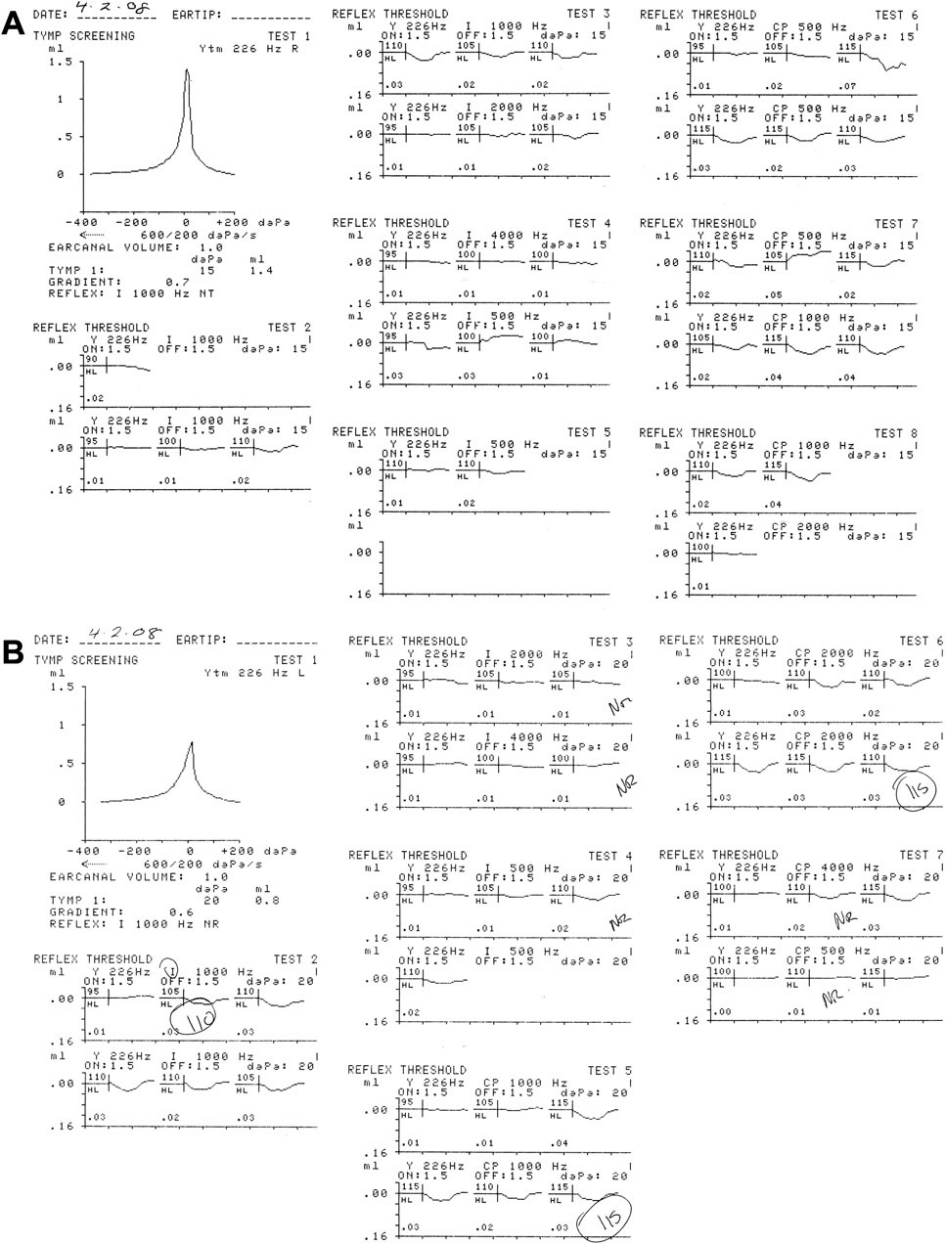
Measurement stapedial reflex thresholds. This image is the tympanogram and ipsilateral (**A**) and contralateral (**B**) stapedial reflex threshold recordings of patient IV:1 illustrating elevated and/or absent reflexes.

**Figure 8:**
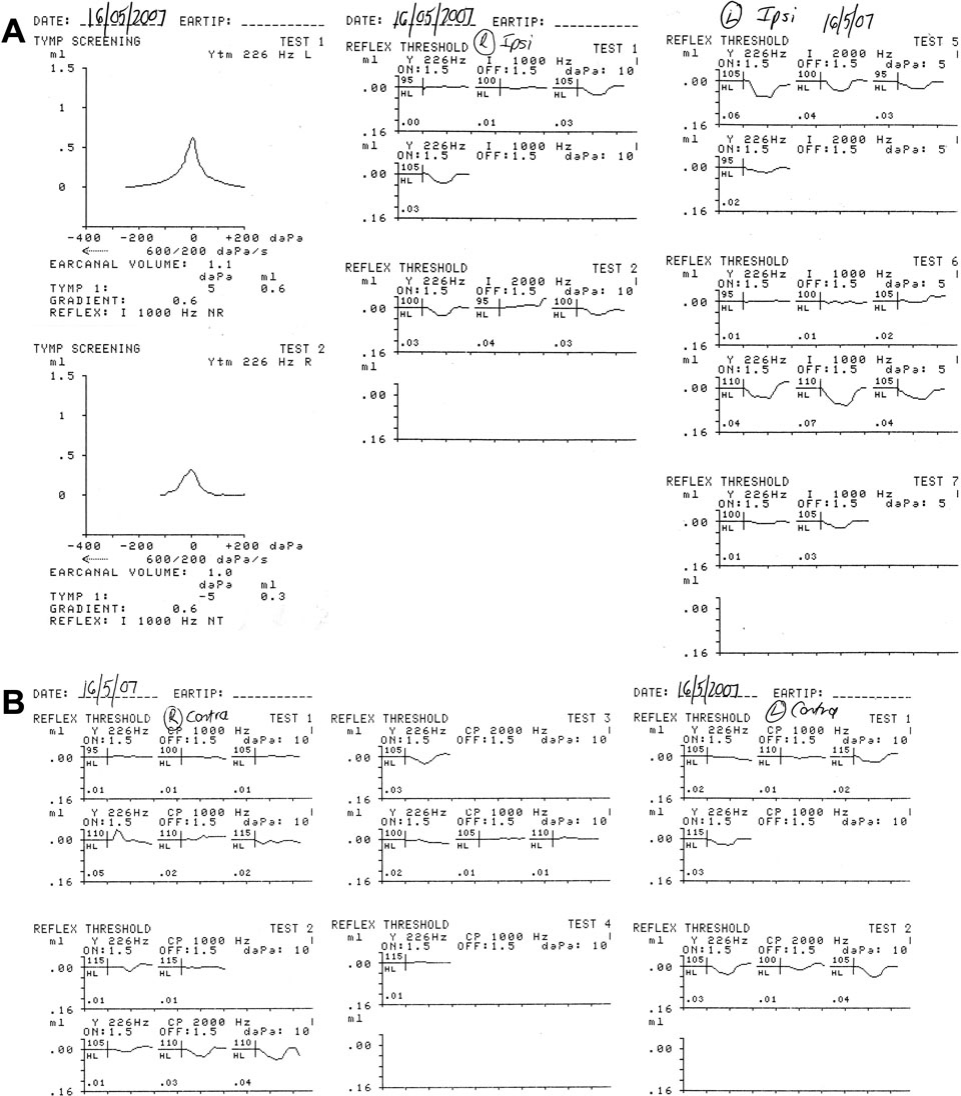
Measurement stapedial reflex thresholds. This image is the tympanogram and ipsilateral (**A**) and contralateral (**B**) stapedial reflex threshold recordings of patient IV:2 illustrating elevated and/or absent reflexes.

**Figure 9:**
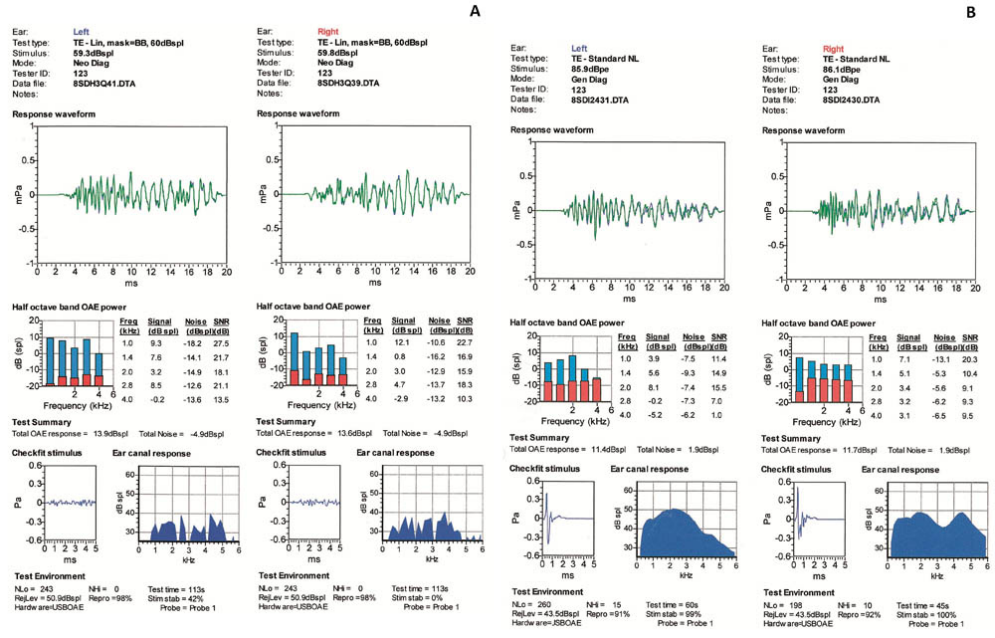
Transient evoked otoacoustic emissions. Transient otoacoustic emission recordings from each ear in patient IV:1 (**A**) and patient IV:2 (**B**) to illustrate normal cochlear outer hair cell function.

**Figure 10:**
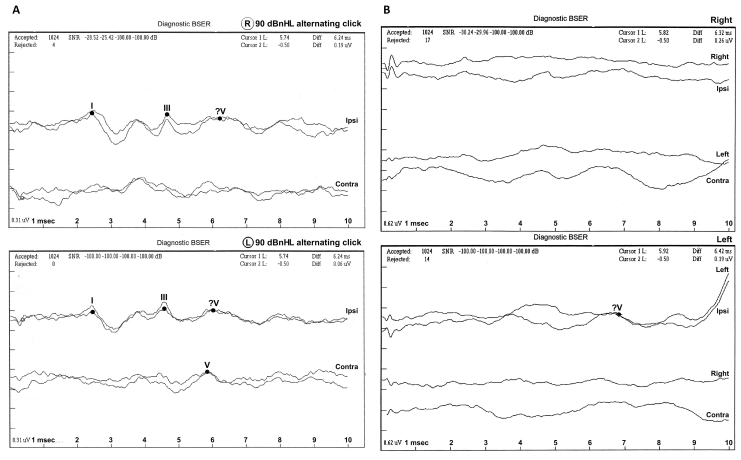
Auditory brainstem-evoked responses. **A**: Auditory brainstem-evoked responses for patient IV:1 (right ear upper section and left ear lower section). **B**: Auditory brainstem-evoked responses for patient IV:2 (right ear upper section and left ear lower section). In patient IV:1 the responses were of poor morphology ipsilaterally. Recording contralaterally, stimulating the left ear the response morphology was markedly abnormal and stimulating the right ear the response was absent. In patient IV:2 all responses were absent. Abbreviations: ipsi represents ipsilateral recording; contra represents contralateral.

Audiologically, neither child complained of any auditory or vestibular symptoms. Patient IV:1 showed a very mild low frequency loss of auditory sensitivity in both ears on pure tone audiometry when first tested at the age of 17 years, and this minor loss progressed to involve the 500–4,000 Hz frequencies in the right ear and the 8,000 Hz frequency in the left ear, 1 year later (Figure 5A). Patient IV:2 showed a 25 dB loss at 8 kHz in the left ear on three audiograms between the ages of 15 and 16 years (Figure 5B). Neither sibling demonstrated a conductive loss, as judged by a masked bone conduction threshold at 500 Hz in patient IV:1 and an unmasked bone conduction threshold at 2,000 Hz in patient IV:2 (Figure 6A,B). In both siblings, impedance studies revealed normal tympanic membrane compliance and middle ear pressures, but stapedial reflex thresholds, recording both ipsilaterally and contralaterally from each ear, were absent in patient IV:1 (Figure 7A,B) and elevated or absent in patient IV:2 (Figure 8A,B). Transient evoked otoacoustic emissions were normal in both children (Figure 9A,B), but brainstem-evoked responses were abnormal. In patient IV:1, the responses were of poor morphology ipsilaterally (Figure 10A), although waves I and III were present and of normal latencies (Table 2) but wave V could not be well defined. Recording contralaterally, stimulating the left ear the response morphology was markedly abnormal and stimulating the right ear the response was absent. In patient IV:2, all responses were absent (Figure 10B).

**Table 2:** Brainstem evoked wave latencies and interwave intervals for siblings and normal departmental ranges (mean±2SD).

**Patient**	**Wave 1**		**Wave III**		**Wave V**		**I-III**		**III-V**		**I-V**	
**R**	**L**	**R**	**L**	**R**	**L**	**R**	**L**	**R**	**L**	**R**	**L**
Sister IL	1.8	1.54	3.82	3.62	~5.24	~5.12	2.02	2.08	~1.96	1.6	~3.98	3.68
Normal range	1.3–1.9		3.3–4.1		5.2–6.0		1.6–2.4		1.4–2.2		3.6–4.4	
Brother IL	Absent						-	-	-	-	-	-

Abbreviations: R represents right; L represents left, IL represents ipsilateral recording.

### Genetic linkage studies

**Figure 11:**
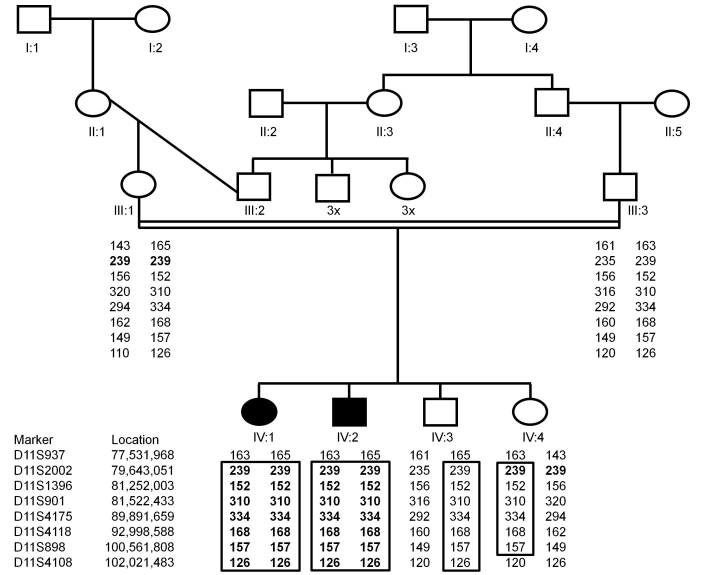
Pedigree diagram and haplotype analysis of the Algerian family with autosomal recessive optic atrophy and auditory neuropathy. The genotyping of microsatellite markers on chromosome 11q14.1–11q22.3 (localization of markers according to NCBI build 36.3) shows a common haplotype indicated by the black framed boxes in the two affected members (IV:1 and IV:2). Open squares represent unaffected males, open circles represent unaffected females, solid squares represent affected males, solid circles represent affected females, and the double line represent intermarriage.

Genome-wide genotyping using the Affymetrix 250 k SNP microarrays revealed four extended regions of homozygosity (>2 Mb; on chromosomes 10 [2 Mb; from rs3127234 to rs912889], 11 [24.17 Mb; from rs10793396 to rs10895556], 16 [15.16 Mb; from rs17839519 to rs7203695], and 19 [2.18 Mb; from rs670091 to rs1654348]) shared at least by the two affected siblings. For chromosome 16, all four children showed an almost complete identical homozygous region of 116 SNPs from 31.63 Mb to 46.79 Mb, including the centromere. Since similar haplotypes could be detected frequently in individuals with other phenotypes and with different ethnic backgrounds, it was assumed that this was unlikely to be a specific finding, and the chromosome 16 candidate region was not further analyzed. Additional genotyping was then performed in all family members using microsatellite marker analysis. Linkage to the regions on chromosome 10 and 19 was excluded by the finding of heterozygous alleles in affected individuals (data not shown). However, genotyping of microsatellite markers within the candidate region at 11q14.1–11q22.3 confirmed that affected individuals were homozygous and unaffected siblings were heterozygous (Figure 11).

### Mutation analysis of the candidate gene

**Figure 12:**
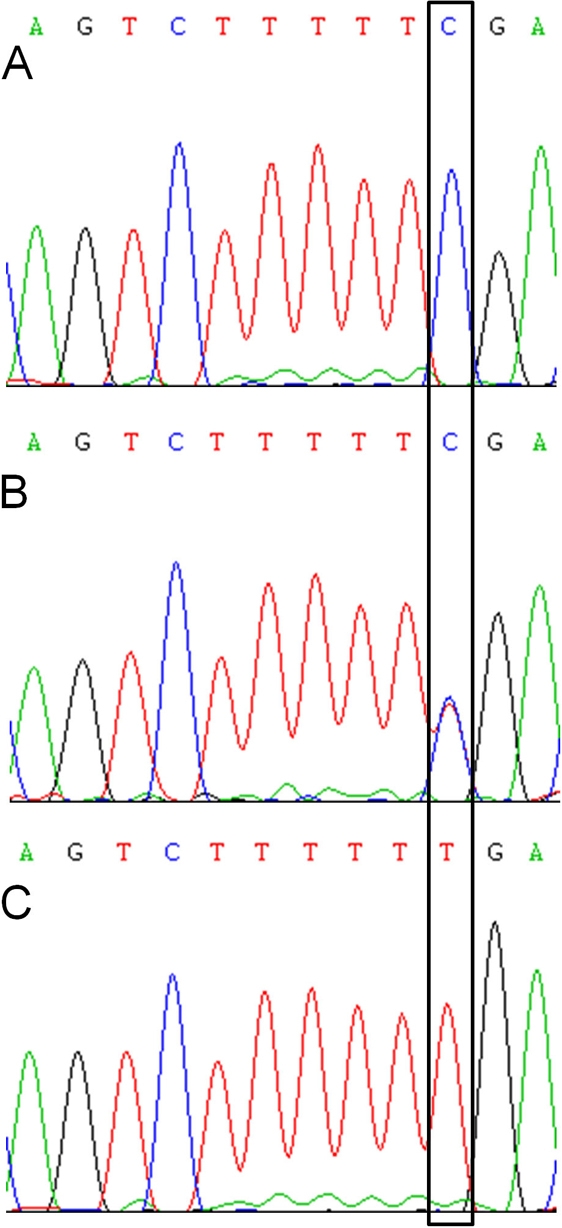
Sequence chromatogram of the transmembrane protein 126A gene mutation in the optic atrophy family, and their corresponding normal sequence. **A**: This panel shows the chromatogram of a control sample with wild-type allele. **B**: This panel shows the chromatogram of the mother (III:1) with the heterozygous transmembrane protein 126A gene (*TMEM126A*) mutation (c.163C>T). **C**: This panel shows the chromatogram of an affected individual (IV:1) with the homozygous *TMEM126A* variant (c.163T). The black framed box indicates the mutation position.

The 24.17 Mb chromosomal region at 11q14.1–11q22.3 contained 175 known genes, pseudogenes, and hypothetical proteins. Interestingly, the *TMEM126A* gene that was recently described in association with autosomal recessive optic atrophy was located within this candidate interval. Therefore, direct sequencing of *TMEM126A* was undertaken, and a nonsense mutation was detected in exon 3 of this gene (c.163C>T; p.Arg55X; Figure 12). The mutation co-segregated with the disease phenotype and was found to be homozygous in all affected individuals and heterozygous in both parents. Previously, this mutation was not detected in 700 control chromosomes [4].

## Discussion

We identified a homozygous nonsense mutation (c.163C>T; p.Arg55X) in *TMEM126A* in an Algerian family with recessive optic atrophy. The same mutation was identified previously in four North African families (one Algerian, one Tunisian, and two Moroccan) with a similar ocular phenotype (haplotyping was consistent with a founder mutation originating ~2,400 years ago [~80 generations]) [4]. However, no further mutations were identified in a cohort of 48 patients with nonsyndromic optic atrophy (in whom mutations in optic atrophy 1 gene [*OPA1*] and the most frequent LHON mutations [mitochondrial DNA {mtDNA} G11778A, G3460A, T14484C, and G15257] had been excluded) [4]. To date, all patients with *TMEM126A* mutations, including the cases reported herein, are of Maghrebian origin and all carry an identical mutation.

The families described by Hanein et al. [4] were characterized by early-onset severe visual impairment, optic disc pallor, and central scotomata. The oldest affected patient lost peripheral visual field function between the ages of 30 and 37. One patient also had moderate hypertrophic cardiomyopathy, and another displayed mild hearing loss and minor brain alterations on magnetic resonance imaging (homogeneous punctate hyperintensities in the stratum subependymale).

Our findings suggest that auditory neuropathy may be a key feature of *TMEM126A*-associated optic atrophy. Both siblings demonstrated a very mild but progressive sensorineural hearing loss, with no evidence of a conductive loss, and normal cochlear function, as judged by normal otoacoustic emissions; but both demonstrated abnormal retrocochlear function, with inner hair cell/neural involvement, as judged by abnormal stapedial reflex thresholds and brainstem-evoked responses. The site of the neural lesion may lie in the functional unit comprised of inner hair cells, the primary afferents (spiral ganglion neurons), and/or the first order synapses between hair cells and the cochlear nerve.

*TMEM126A* encodes a mitochondrial protein found in higher eukaryotes [4]. Laboratory analysis of respiratory chain function in patients with homozygous *TMEM126A* mutations has not shown consistent abnormalities (although one patient demonstrated partial deficiency of Complex I). Retinal ganglion cells are located in the inner retina and their axons remain unmyelinated until they exit the globe and are organized in bundles to form the optic nerve. During their intraocular path, the unmyelinated axons are very energy dependent (to transmit the action potential) and therefore vulnerable in disorders of mitochondrial function [16]. Hence, optic atrophy is a common feature of mitochondrial disorders, and two other nonsyndromic causes of optic atrophy, LHON and autosomal dominant optic atrophy Kjer type, are caused, respectively, by mutations in mitochondrial DNA and the nuclear gene *OPA1* that encodes a mitochondrial protein. In addition, optic atrophy is a prominent feature of many other neurodegenerative diseases caused by primary mitochondrial dysfunction [16]. Hearing loss is a common feature in mitochondrial disease, although frequently cochlear dysfunction is reported and auditory neuropathy is considered a rare finding [5].

Interestingly, mutations in *OPA1*, although originally described in nonsyndromic hereditary optic neuropathy, have recently been reported to also cause a syndromic form of optic atrophy associated with sensorineural deafness, ataxia, and multiple mitochondrial DNA deletions [17,18]. The hearing loss is reported to be suggestive of an auditory neuropathy, while in Wolfram Syndrome or diabetes insipidus and mellitus with optic atrophy and deafness (DIDMOAD), a recent clinicopathological study reported cochlear histopathological abnormalities with loss of the organ of Corti in the basal turn of the cochlea and mild focal atrophy of the stria vascularis [19].

Our findings suggest that a diagnosis of autosomal recessive *TMEM126A*-associated optic atrophy and auditory neuropathy (ARTOAN) should be considered in patients with optic atrophy and deafness. Furthermore, patients with homozygous *TMEM126A* mutations should be investigated for subclinical evidence of auditory neuropathy.
